# Supplementary material for: Discovery of Novel STING Inhibitors Based on the Structure of the Mouse STING Agonist DMXAA
Source: Molecules. 2023 Mar 23;28(7):2906. doi: 10.3390/molecules28072906 (PMC10095708; doi:10.3390/molecules28072906)
Supplement: Supplementary file 1 [file molecules-28-02906-s001.zip › molecules-2278643-supplementary.pdf]

Supplementary Material

Table S1. The pocket properties of the STING crystal structures.

Species	PDB code	Ligand	Binding-site surface (Å^2) [a]	Surface-Volume-Ratio [a]	Binding-site Volume (Å^3) [a]
**Mouse**	4KCO	none	479.50	1.62	296.45
	4LOJ	cGAMP	499.45	1.75	286.21
	4LOL	DMXAA	840.76	2.83	297.47
	4JC5	CMA	545.47	1.88	290.14
**Human**	4EMU	none	1955.73	2.28	858.62
	4LOH	cGAMP	588.85	1.95	301.57
	6UKV	MSA-02	426.61	1.44	296.96
	6XNP	SR717	472.64	1.66	285.18

[a] Calculated the above data collection by the GeoMine module of ProteinPlus (https://proteins.plus).

Table S2. Docking scoring of compound **11** with different conformations.

conformation	docking score	glide emodel	dG Bind
**1**	-6.104	-44.532	-52.36
**2**	-6.147	-43.547	-32.63
**3**	-6.274	-43.395	-51.00
**4**	-5.933	-42.595	-43.86
**5**	-5.809	-41.833	-48.18
**6**	-5.869	-40.511	-44.94
**7**	-5.034	-40.305	-50.88
**8**	-5.285	-39.637	-31.28
**9**	-5.679	-39.397	-50.72
**10**	-4.760	-39.210	-44.73
**11**	-4.868	-38.995	-35.27
**12**	-4.734	-38.561	-35.43
**13**	-5.202	-38.329	-52.32
**14**	-4.904	-38.087	-34.87
**15**	-4.777	-37.384	-39.99
**16**	-4.138	-34.221	-44.66

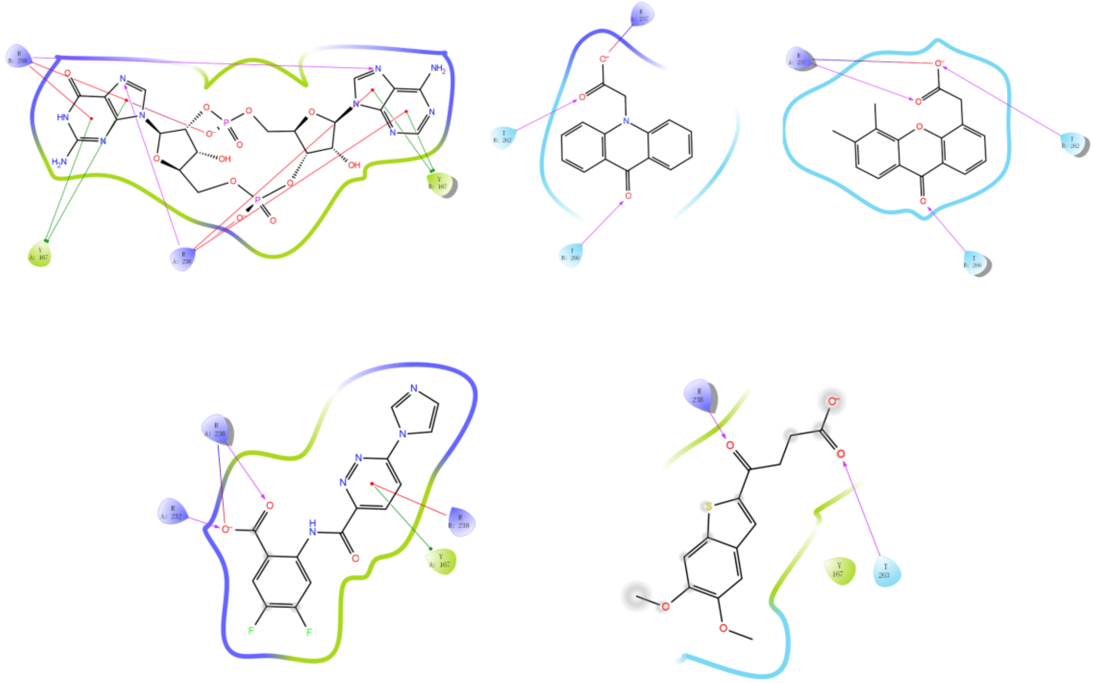

Figure S1. Protein−ligand interaction diagram for the STING complexed with agonists (5.0 Å around the ligand). Including PDB ID codes: 4LOH, 4JC5, 4LOL, 6XNP, and 6UKV.

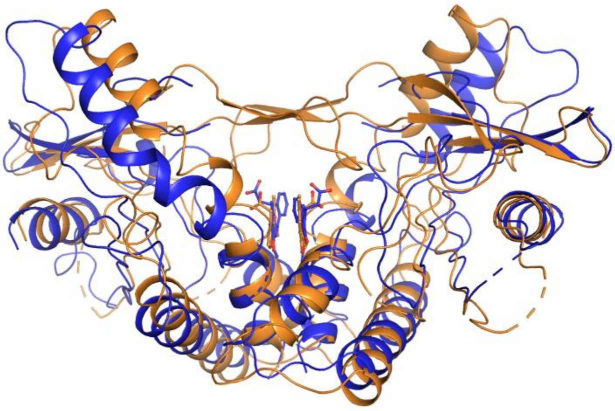

Figure S2. Superposition of the structure of compound 11 bound to hSTING (shown as dark blue) with the structure of DMXAA bound to mSTING (PDB ID 4LOL, shown as orange).

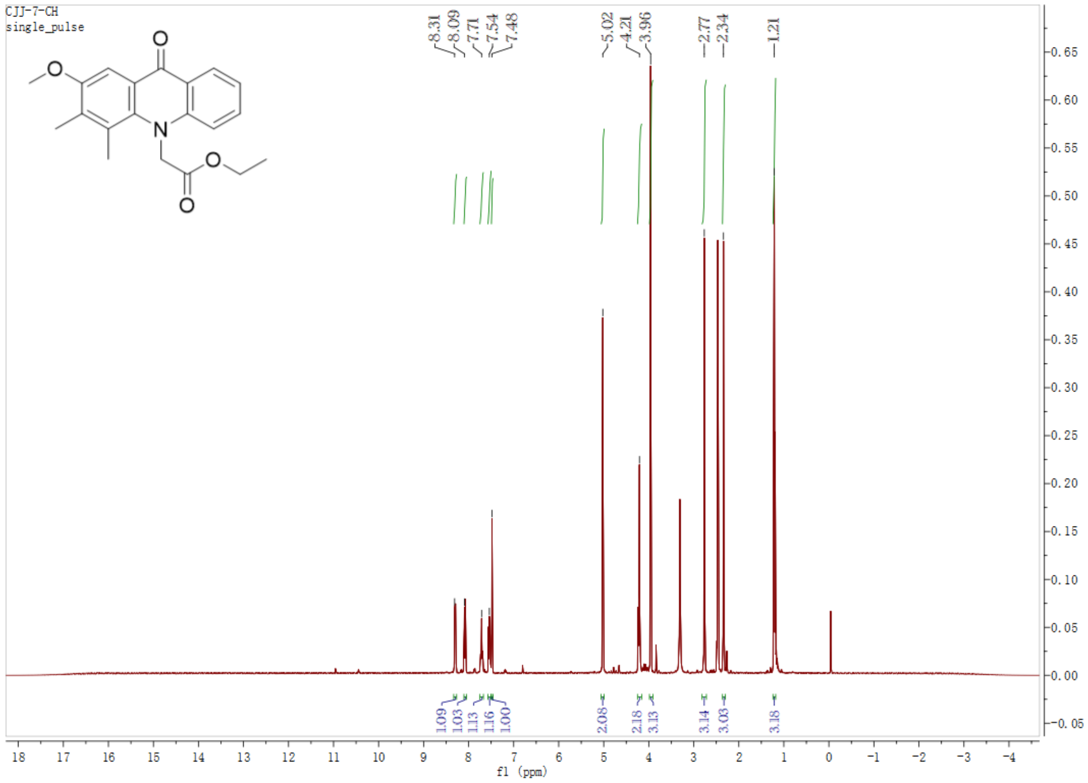

Figure S3. ^{1}H NMR spectra of compound **10**.

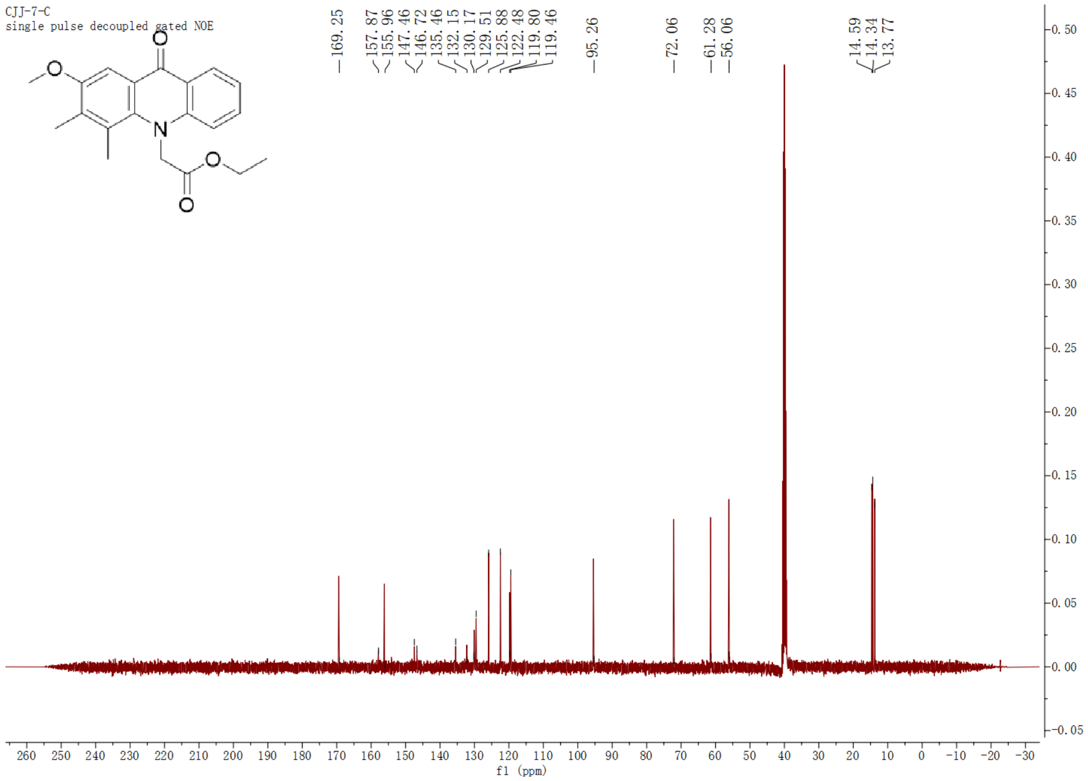

Figure S4. ^{13}C NMR spectra of compound **10**.

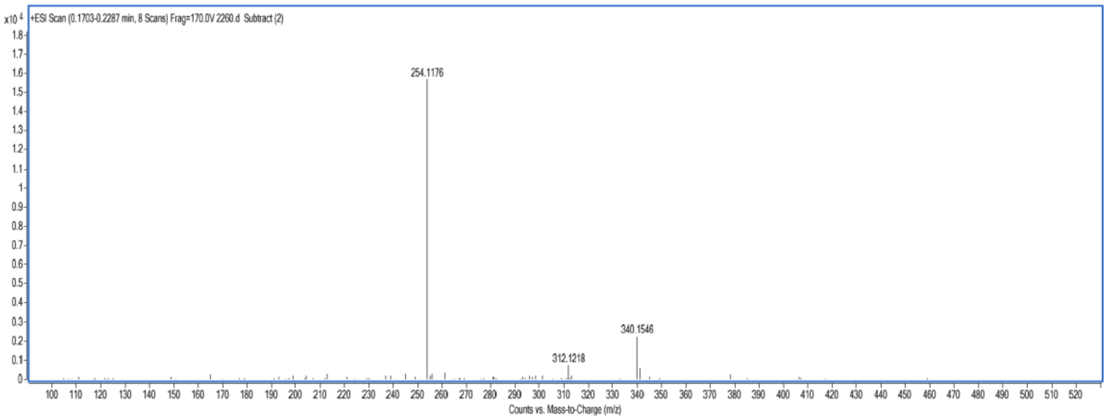

Figure S5. HRMS spectra of compound **10**.

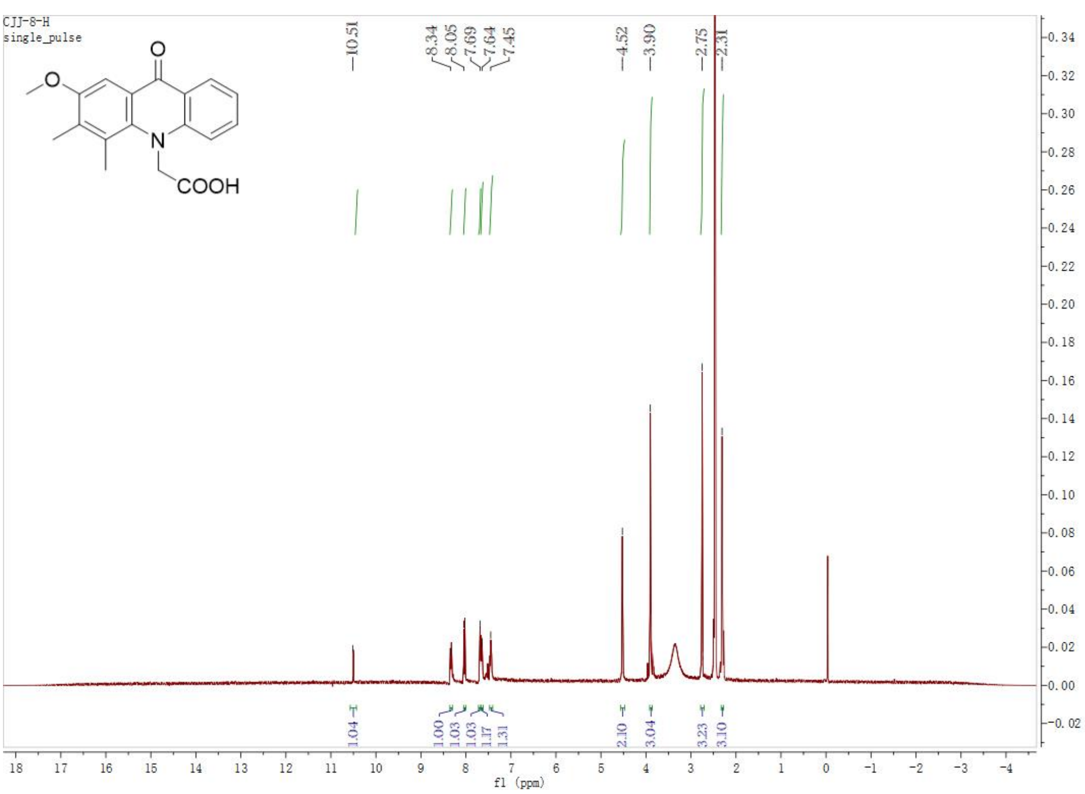

Figure S6. ^{1}H NMR spectra of compound **11**.

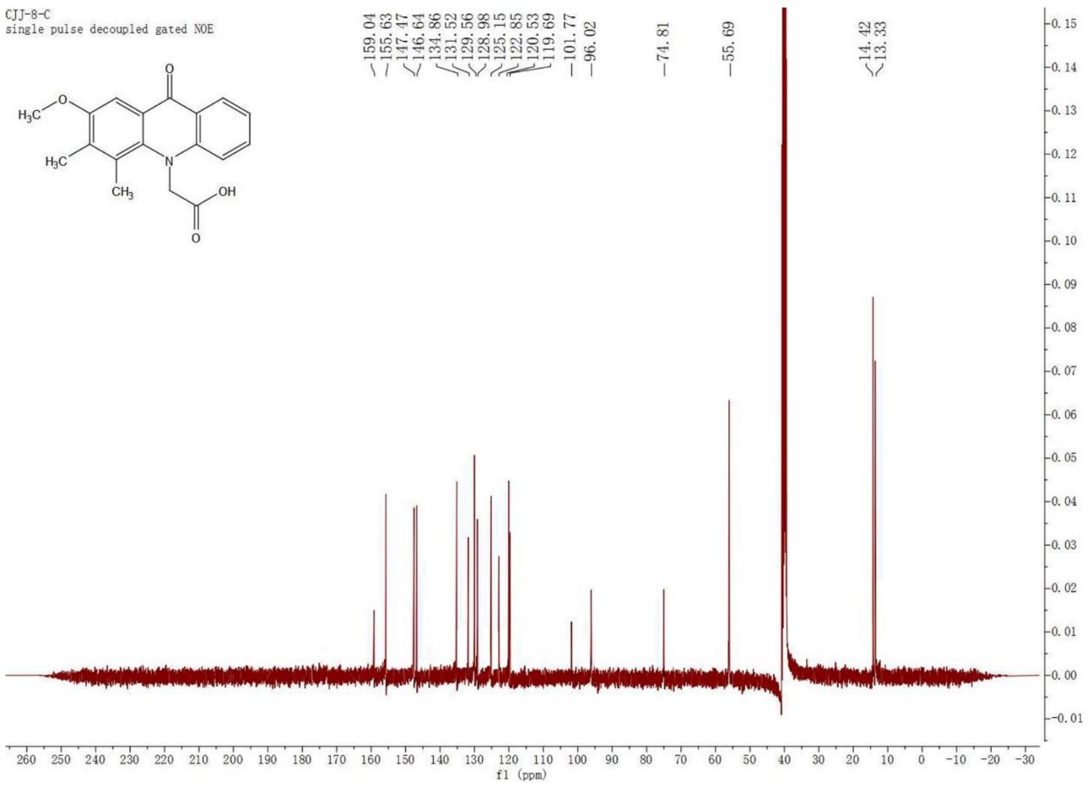

Figure S7. ^{13}C NMR spectra of compound **11**.

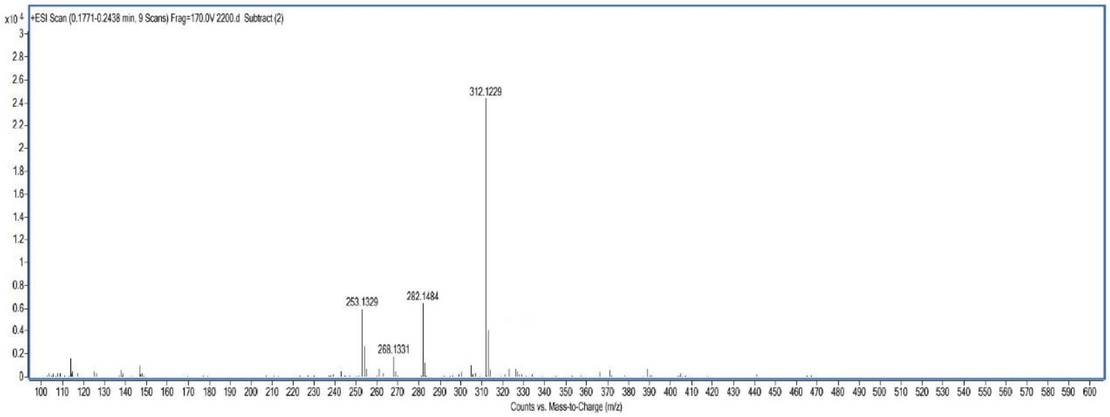

Figure S8. HRMS spectra of compound **11**.

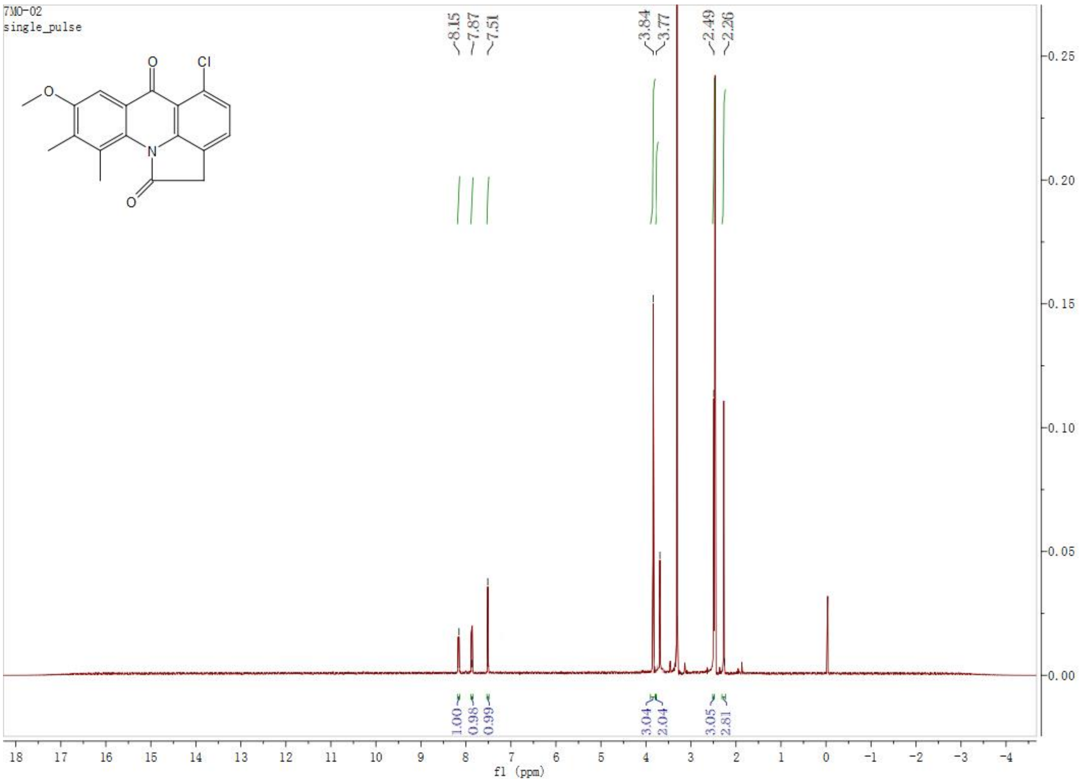

Figure S9. ^{1}H NMR spectra of compound **25**.

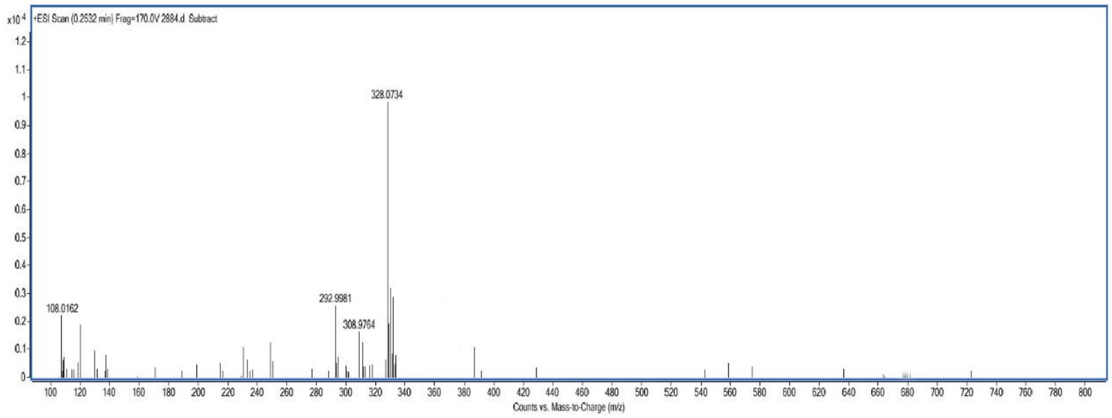

Figure S10. HRMS spectra of compound **25**.

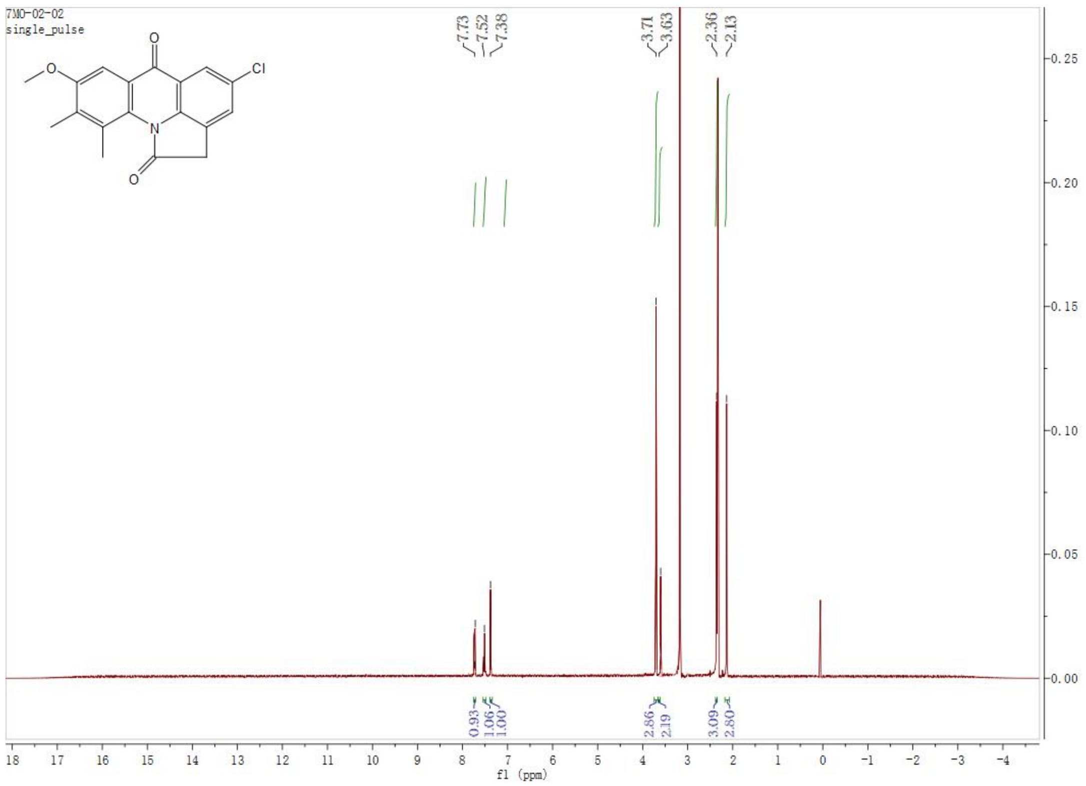

Figure S11. ^1H NMR spectra of compound **26**.

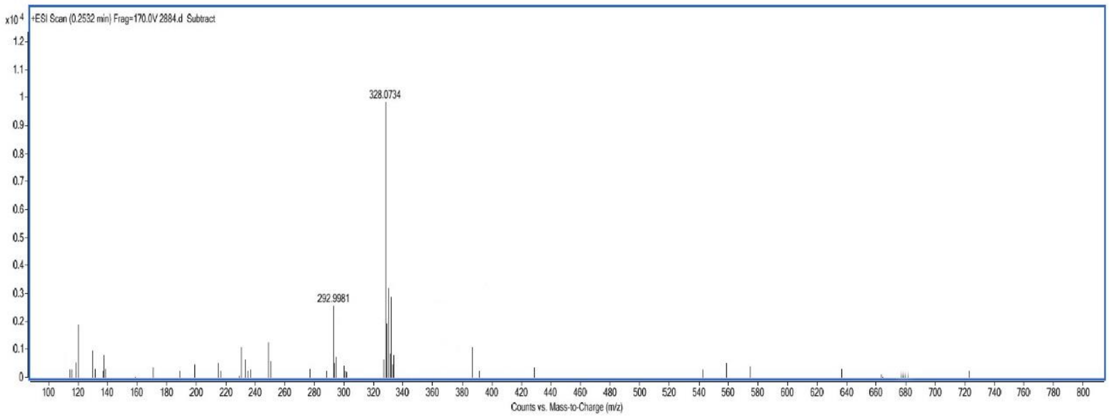

Figure S12. HRMS spectra of compound **26**.

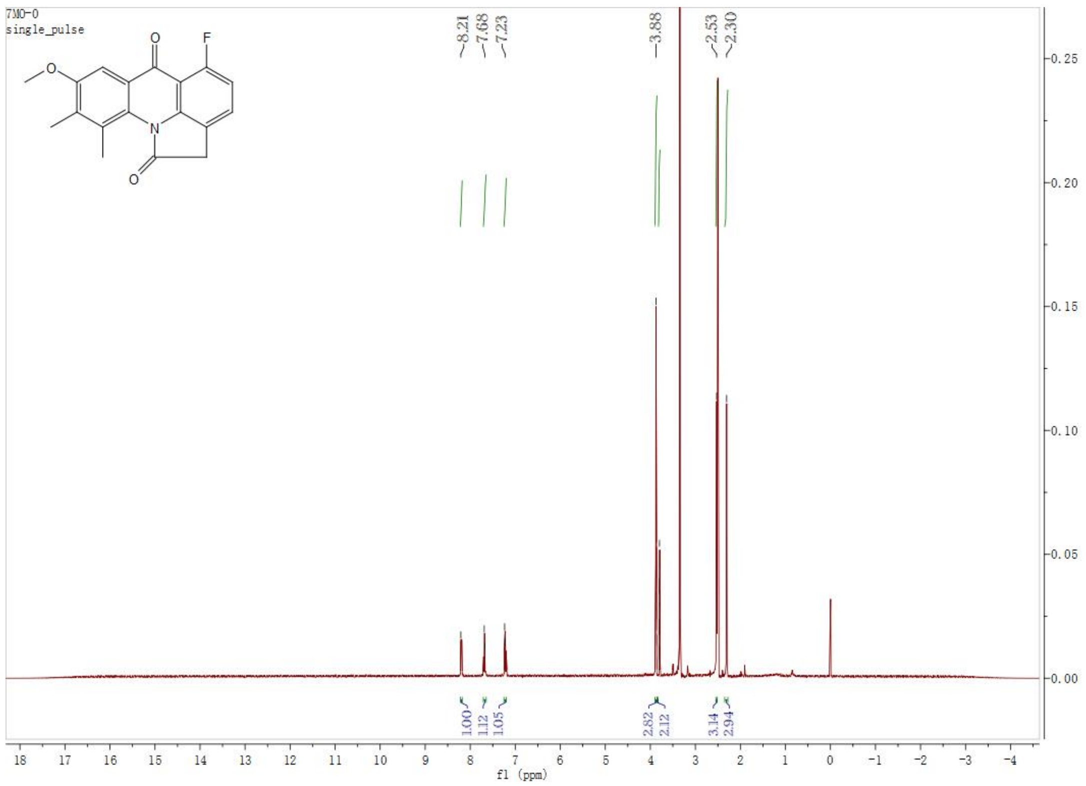

Figure S13. ^{1}H NMR spectra of compound **27**.

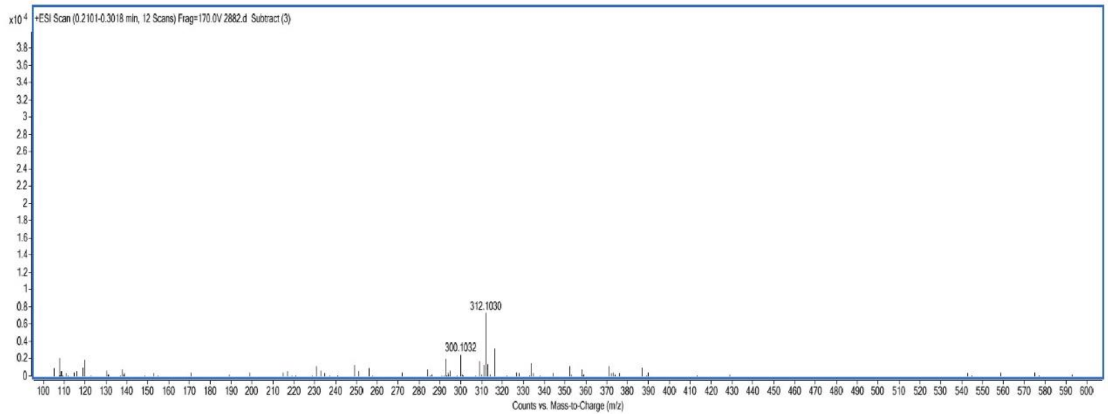

Figure S14. HRMS spectra of compound **27**.

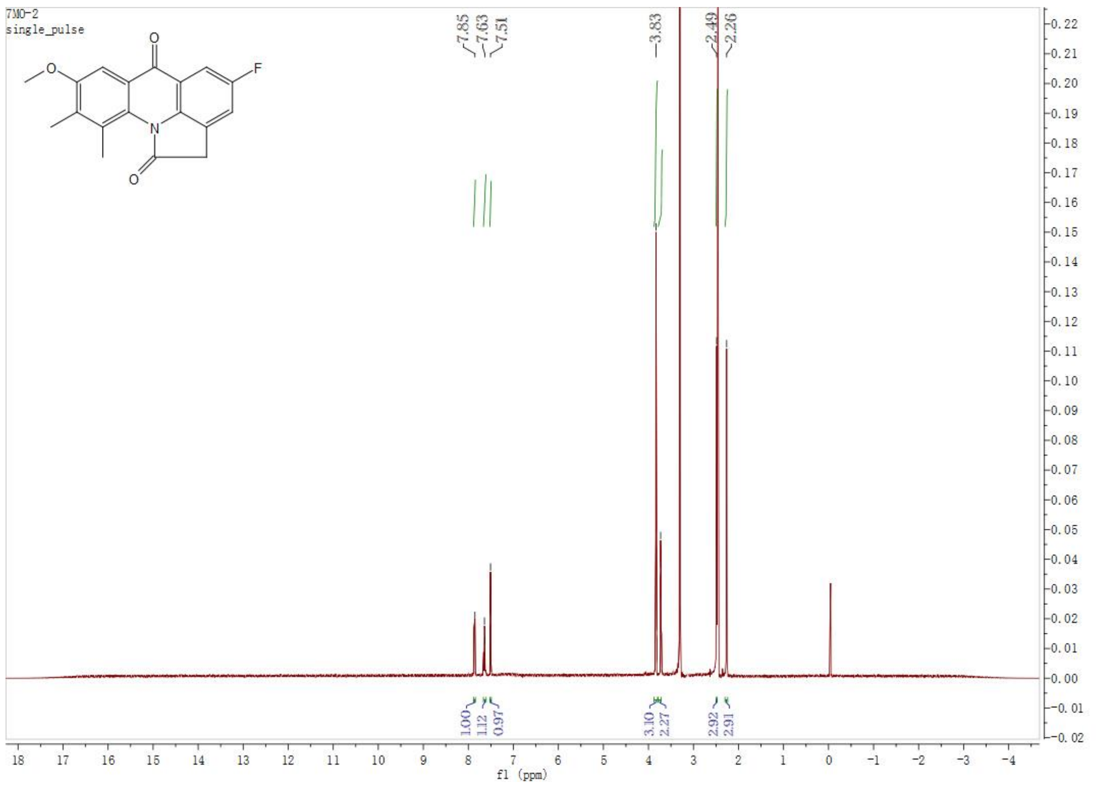

Figure S15. ^{1}H NMR spectra of compound **28**.

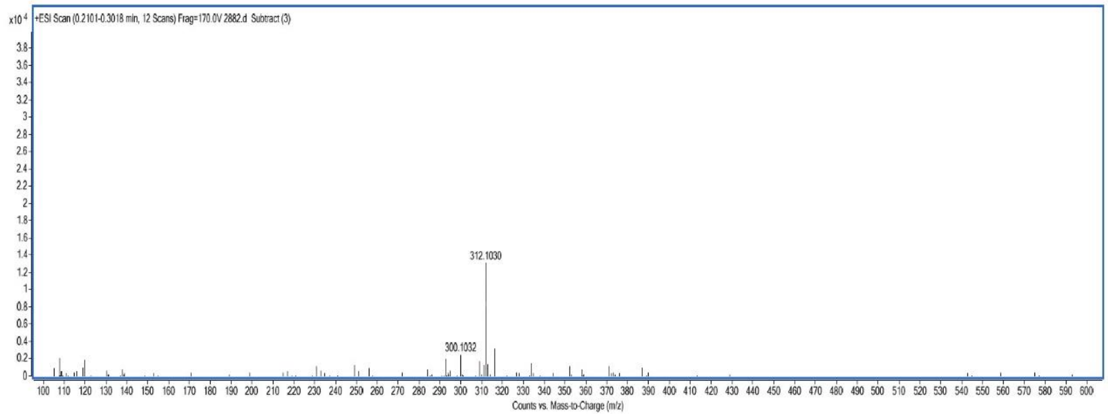

Figure S16. HRMS spectra of compound **28**.

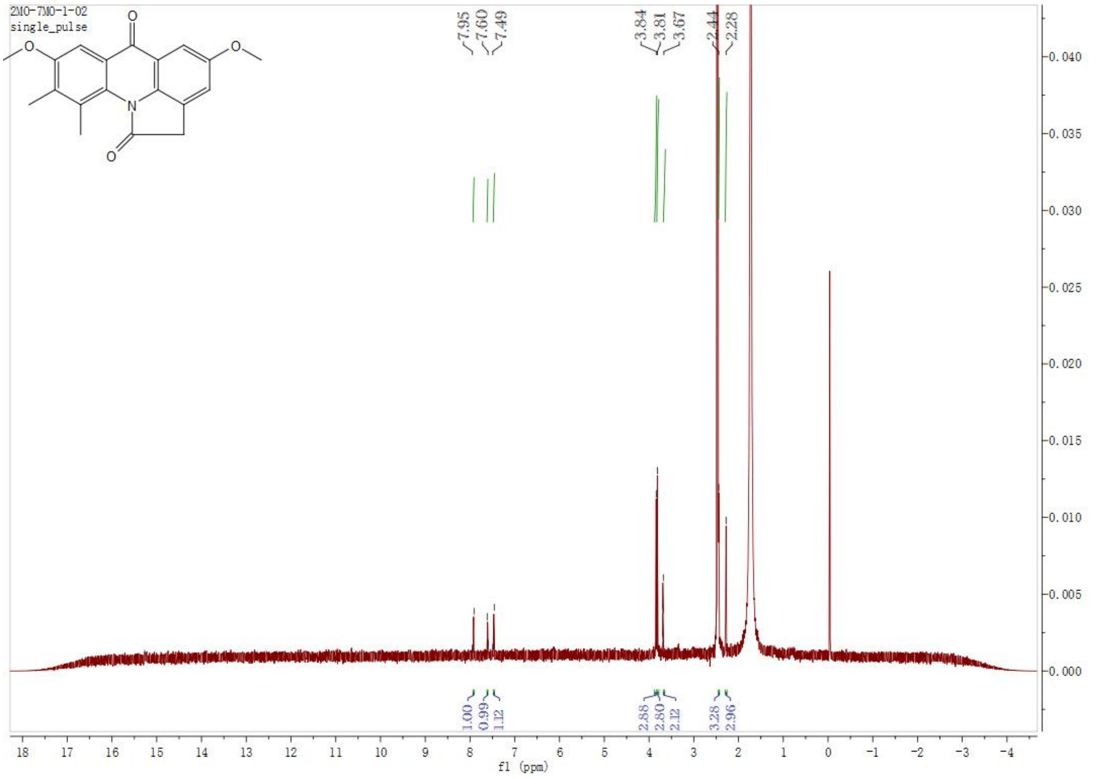

Figure S17. ^{1}H NMR spectra of compound **29**.

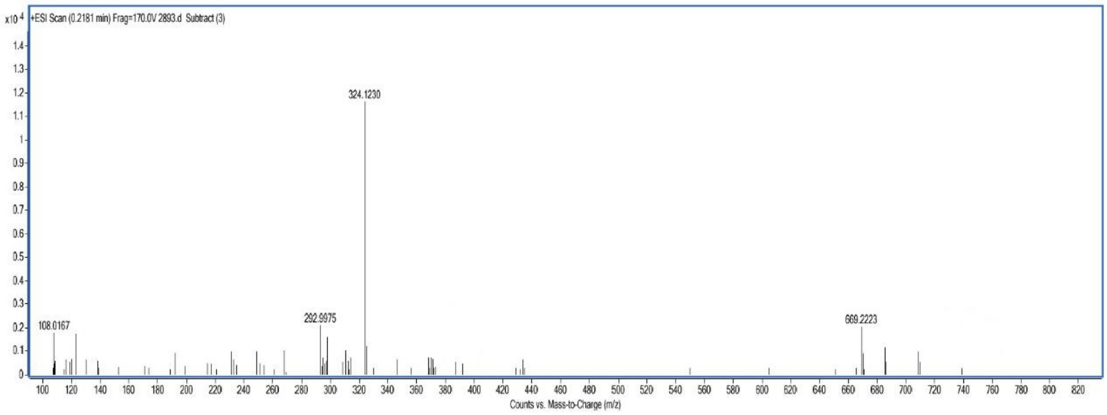

Figure S18. HRMS spectra of compound **29**.

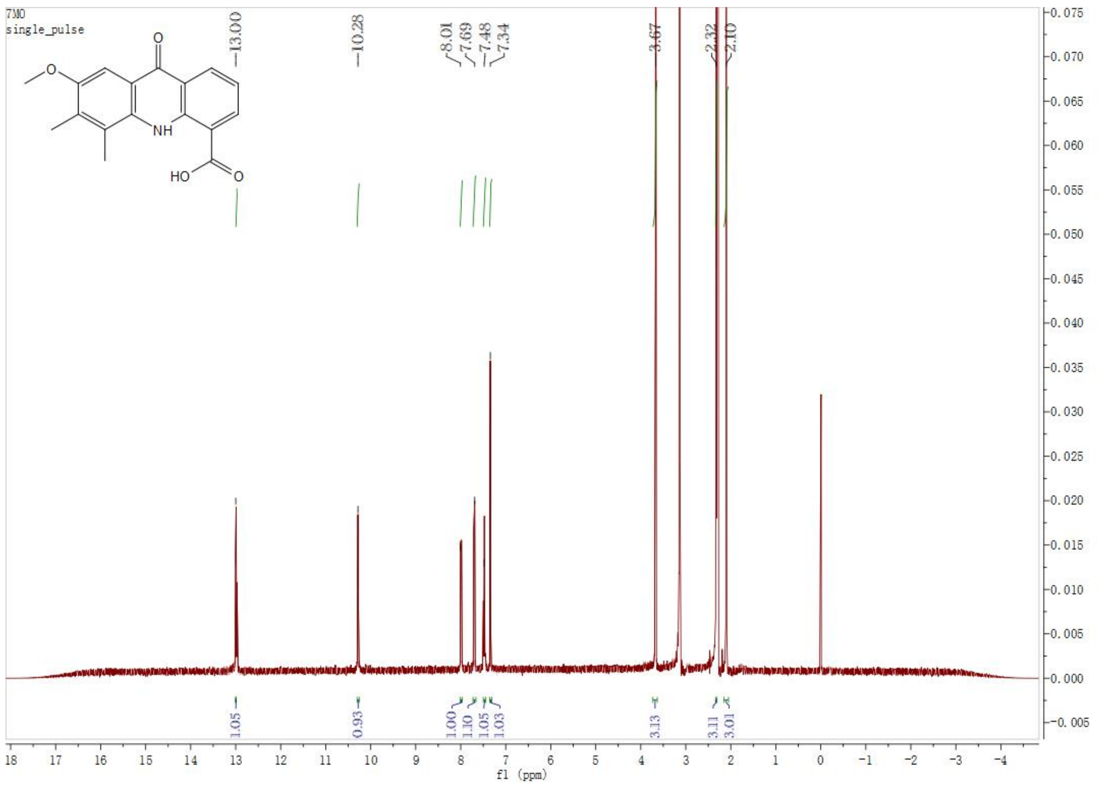

Figure S19. ^{1}H NMR spectra of compound **33**.

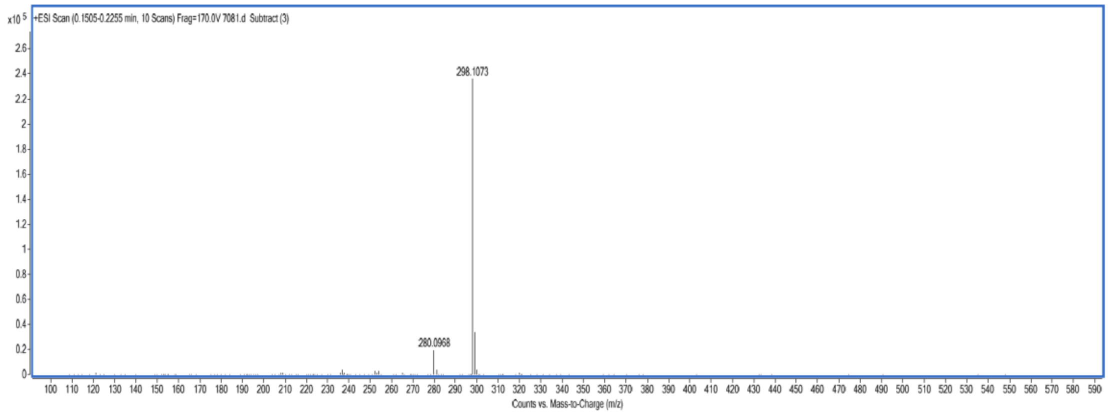

Figure S20. HRMS spectra of compound **33**.

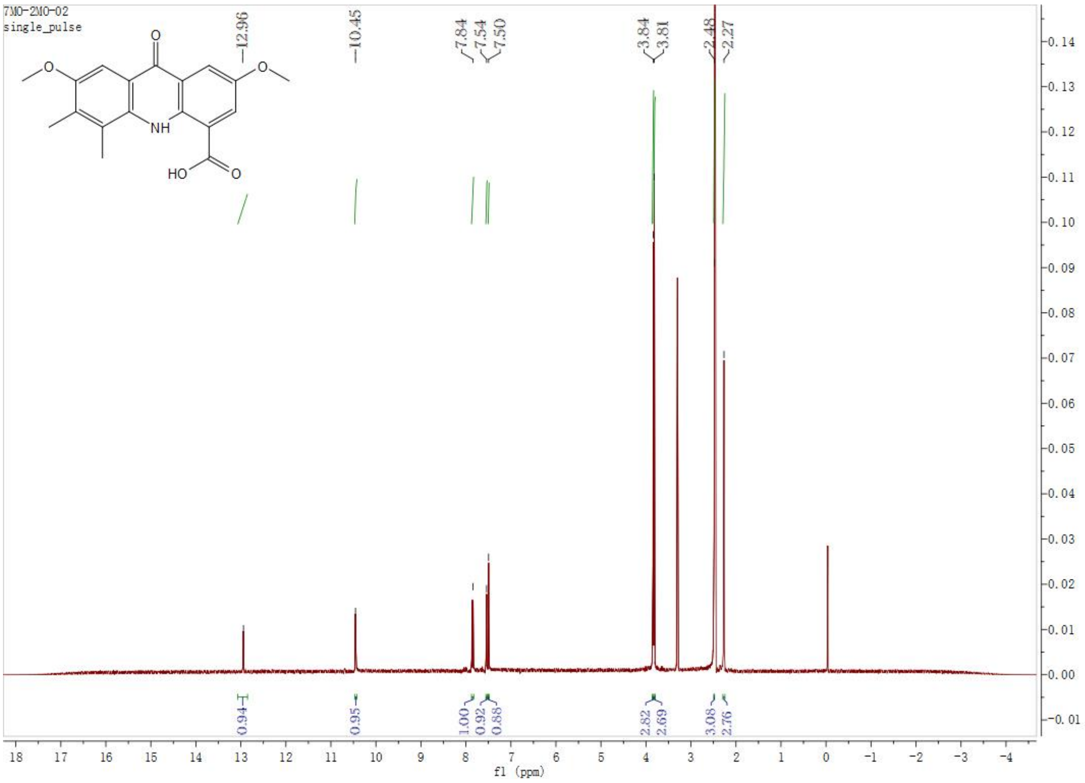

Figure S21. ^{1}H NMR spectra of compound **34**.

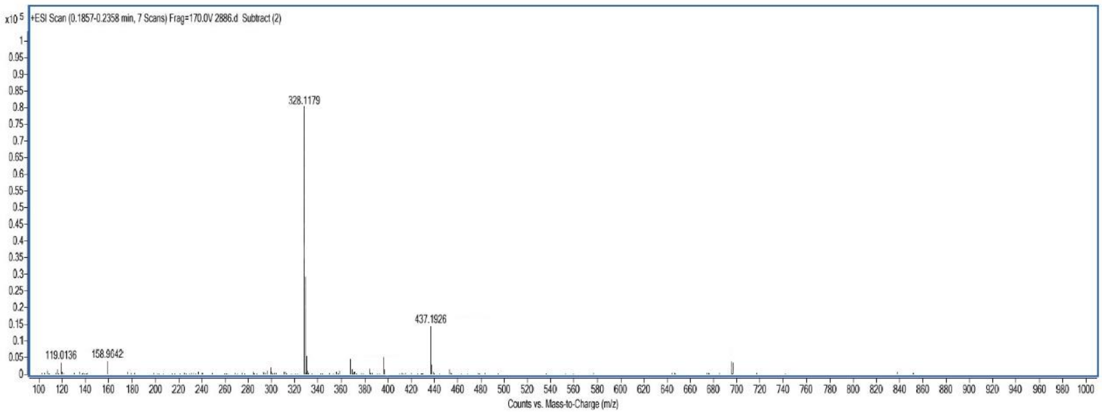

Figure S22. HRMS spectra of compound **34**.
